# Down-Regulation of AKT Signalling by Ursolic Acid Induces Intrinsic Apoptosis and Sensitization to Doxorubicin in Soft Tissue Sarcoma

**DOI:** 10.1371/journal.pone.0155946

**Published:** 2016-05-24

**Authors:** Victor Hugo Villar, Oliver Vögler, Francisca Barceló, Javier Martín-Broto, Jordi Martínez-Serra, Valentina Ruiz-Gutiérrez, Regina Alemany

**Affiliations:** 1 Group of Clinical and Translational Research, Department of Biology, Institut Universitari d’Investigacions en Ciències de la Salut (IUNICS), University of the Balearic Islands, Palma de Mallorca, Spain; 2 Department of Oncology, University Hospital Virgen del Rocío and Biomedicine Institute of Sevilla (IBIS), Sevilla, Spain; 3 Department of Hematology, University Hospital Son Espases, Palma de Mallorca, Spain; 4 Instituto de la Grasa, Consejo Superior de Investigaciones Científicas (CSIC), Sevilla, Spain; 5 CIBER:CB06/03 Fisiopatología de la Obesidad y la Nutrición, CIBERobn, Instituto de Salud, Carlos III (ISCIII), Spain; Henry Ford Health System, UNITED STATES

## Abstract

Several important biological activities have been attributed to the pentacyclic triterpene ursolic acid (UA), being its antitumoral effect extensively studied in human adenocarcinomas. In this work, we focused on the efficacy and molecular mechanisms involved in the antitumoral effects of UA, as single agent or combined with doxorubicin (DXR), in human soft tissue sarcoma cells. UA (5–50 μM) strongly inhibited (up to 80%) the viability of STS cells at 24 h and its proliferation in soft agar, with higher concentrations increasing apoptotic death up to 30%. UA treatment (6–9 h) strongly blocked the survival AKT/GSK3β/β-catenin signalling pathway, which led to a concomitant reduction of the anti-apoptotic proteins c-Myc and p21, altogether resulting in the activation of intrinsic apoptosis. Interestingly, UA at low concentrations (10–15 μM) enhanced the antitumoral effects of DXR by up to 2-fold, while in parallel inhibiting DXR-induced AKT activation and p21 expression, two proteins implicated in antitumoral drug resistance and cell survival. In conclusion, UA is able to induce intrinsic apoptosis in human STS cells and also to sensitize these cells to DXR by blocking the AKT signalling pathway. Therefore, UA may have beneficial effects, if used as nutraceutical adjuvant during standard chemotherapy treatment of STS.

## Introduction

The consumption of certain fruits and vegetables of the traditional Mediterranean diet has been associated with low incidence of cancer [1, 2], providing evidence that certain bioactive dietary components of this diet have a great potential in cancer prevention or treatment. Of particular interest in this context have been various fruits, including olive fruits (*Olea europaea*), cranberries, apples, pears and prunes, and some medicinal herbs, such as lavender and salvia, all of them containing the natural pentacyclic triterpene ursolic acid (UA) [3, 4]. This natural compound has received increasing attention in the last few years due to its antitumoral effect, exhibiting antiproliferative and apoptotic activities in a variety of human carcinoma cells [5]. Several molecular mechanisms have been implicated in UA-induced cell cycle arrest and apoptosis, such as inhibition of DNA replication [6], increase of intracellular levels of reactive oxygen species [7], suppression of nuclear factor-kappaB activation [8, 9] as well as down-regulation of anti-apoptotic proteins (e.g., Bcl-2, Bcl-xl and survivin) and caspase activation [10–12]. Moreover, induction of cell differentiation [13], inhibition of angiogenesis and invasion of tumor cells [14, 15] have also been attributed to UA. Interestingly, inactivation of the phosphatidylinositol 3-kinase (PI3K)/AKT signalling pathway was recently found to be involved in some of its above described antitumoral effects [10, 15, 16].

The intracellular PI3K/AKT pathway is an important cell signalling route for growth factor-induced proliferation and survival of several cancer cells [17, 18]. Once activated by PI3K, AKT moves from the cell membrane to the cytoplasm and/or nucleus, where it promotes cell cycle progression and prevents apoptosis in several ways, thereby controlling essential cellular regulator proteins (e.g., cyclin D, c-Myc or β-catenin) and cyclin-dependent kinase (CDK) inhibitors (e.g., p21/WAF1). In this regard, cytosolic stabilization of p21 by its direct phosphorylation may be one of the mechanisms by which AKT contributes to prevent apoptosis in cancer cells [19]. Moreover, AKT activation also inhibits GSK3β through phosphorylation, thereby blocking c-Myc and β-catenin degradation, which in turn facilitates tumor cell proliferation and invasion [20, 21].

Soft tissue sarcoma (STS) constitute a heterogeneous group of malignant mesenchymal tumors, for which the standard chemotherapy, based on doxorubicin (DXR) alone or in combination with other drugs, does not achieve significant improvements in terms of overall survival [22]. Interestingly, immunohistochemical studies found elevated phosphorylation of AKT and a concomitant up-regulation of downstream effectors (e.g., GSK3β and β-catenin) in tumors of patients with STS, especially in synovial sarcoma [23, 24] and leiomyosarcoma [25].

Although many studies evaluated the efficacy of UA in a variety of cell and animal models of adenocarcinoma (for a review see [5]), only little is known regarding its effects in STS and whether a combination of this natural compound with conventional chemotherapy could improve treatment outcome for this type of solid tumors. Our study focused on the efficacy and molecular mechanisms involved in the antitumoral effects of UA, either as individual agent or in combination with the standard chemotherapeutic DXR in STS cells.

## Materials and Methods

### Cell culture and treatments

The human synovial sarcoma SW982 (HTB-93^™^), leiomyosarcoma SK-UT-1 (HTB-114^™^) and fibrosarcoma HT-1080 (CCL-121^™^) cell lines were obtained directly from the American Type Culture Collection (Manassas, VA). These cell lines were chosen because they show low sensitivity to doxorubicin (DXR) treatment [26], which is a prerequisite for the aim of the study. SW982 and SK-UT-1 cells express the multidrug resistant-related protein 1 (MPR-1) at similar levels, but differ in the level of P-glycoprotein (P-gp), which is barely expressed in SK-UT-1 cells. Synovial sarcoma cells were grown in Leibovitz’s L-15 medium (Invitrogen S.A, Barcelona, Spain), whereas leiomyosarcoma SK-UT-1 and fibrosarcoma HT-1080 cells were cultured in DMEM medium (LabClinics S.A, Barcelona, Spain) supplemented with MEM-non essential aminoacids (dilution 1:100) and 1 mM sodium pyruvate. Both media contained 2 mM L-glutamine and were supplemented with 10% (v/v) fetal bovine serum, 100 units/ml penicillin and 100 μg/ml streptomycin. Tissue culture supplements were all purchased from Sigma-Aldrich (Madrid, Spain). When the cells reached 60–70% confluence, vehicle (DMSO), triciribine phosphate (TCN), ursolic acid (UA) or DXR as single agents or their combinations were added to the medium for 6, 9, 12 and 24 h. UA and DXR were purchased from Sigma-Aldrich (Madrid, Spain). Stock solutions of UA and TCN were prepared at a concentration of 10 mM in dimethylsulfoxide (DMSO) or in water in the case of DXR.

### Cell viability

Synovial sarcoma, leiomyosarcoma SK-UT-1 and fibrosarcoma HT-1080 cells were plated in 96-well plates at a density of 11 x 10^3^ or 8 x 10^3^ cells per well in 200 μl of their respective growth medium, and cultured for 24 h. To test the effectiveness of UA on cell viability, cells were exposed to increasing concentrations of UA (1–50 μM) for 24 h. The compound concentration resulting in 50% inhibition of cell viability (IC_50_) was determined using GraphPad software. To determine whether UA or TCN would increase sensitivity of these cell lines to DXR, cells were treated with varying concentrations of DXR (range 0.1–10 μM) with or without UA (10 or 15 μM) or TCN (30 μM). After 24 h, the viability of the cells was measured using the methylthiazoletetrazolium (MTT) method. The mean percentage of cell survival relative to that of vehicle-treated cells was estimated from data of three individual experiments each performed in triplicate.

### Soft agar colony assay

A base layer of 0.6% agarose (agarose D1 Low EEO, Conda, Pronadisa) in complete growth medium was plated in 6-well plates. Then, 20 x 10^3^ cells cells were suspended in 0.3% agarose in the corresponding complete growth medium with or without UA (5–50 μM), placed on top of the base layer and cultured at 37°C. Synovial sarcoma SW982 and leiomyosarcoma SK-UT-1 cells were fixed and stained with 0.005% crystal violet dye at 4°C overnight, after 21 or 7 days, respectively. Images were taken at 40-fold magnification from four different fields from each well, and colonies larger than 50 μm were counted as positives.

### Cell cycle analysis and apoptosis

The apoptotic index and cell cycle analysis were performed on STS cells by flow cytometry as described previously [26]. Cell populations in the different phases of cell cycle (sub-G1 (cell death), G0/G1, S/G2/M peaks) were determined based on their DNA content in a Beckman Coulter Epics XL flow cytometer. Cellular apoptosis was also determined by assessment of the cleavage of caspase 9, 3 and PARP and by measuring the expression of the anti- and pro-apoptotic proteins Bcl-2 and Bax, respectively, by immunoblot analysis.

### Immunoblot analysis

Preparation of cell extracts and protein assays were performed as described previously [26]. Primary polyclonal antibodies anti-phospho-AKT (P-AKT) (Ser473; 60 kDa), anti-AKT (60 kDa), anti-poly ADP-ribose polymerase (anti-PARP) (116 and 89 KDa), anti-phospho-GSK3β (Ser9) (46 KDa), anti-β-Catenin (92 KDa), anti-c-Myc (70–57 KDa), anti-p21 (CIP1/WAF1) (21 kDa), anti-Bax (20 kDa), anti-Bcl 2 (28 kDa), anti-caspase 3 (35 kDa), anti-caspase 9 (47 kDa), anti-cleaved caspase-3 (Asp175) (17–19 kDa), anti-cleaved caspase 9 (Asp330) (17–37 kDa) and anti-β-actin (42 kDa) were obtained from Cell Signaling Technology (Beverly, MA) and diluted 1:1000. IRDye 800CW-conjugated anti-rabbit and anti-mouse secondary antibodies were purchased from Bonsai Technologies (Spain). The intensity of band immunoreactivity was analysed with the Odyssey imaging system from LI-COR.

### Quantification of intracellular doxorubicin

Synovial sarcoma cells were treated with vehicle (DMSO) or DXR (1 μM) in the absence or presence of UA (10 μM) for 2 or 24 h. After this time, cells were harvested, washed and resuspended in PBS at 37°C. Fluorescence attributable to intracellular DXR was immediately measured by flow cytometry (Beckman Coulter Epics XL flow cytometer).

### Data analysis

The results are expressed as the mean ± S.E.M. One-way analysis of variance (ANOVA) followed by Bonferroni´s multiple comparison test was used for statistical evaluations. Differences were considered statistically significant at *P* < 0.05. All calculations were done by GraphPad Software.

## Results

### UA treatment inhibits viability and growth in STS cells

**Fig 1:**
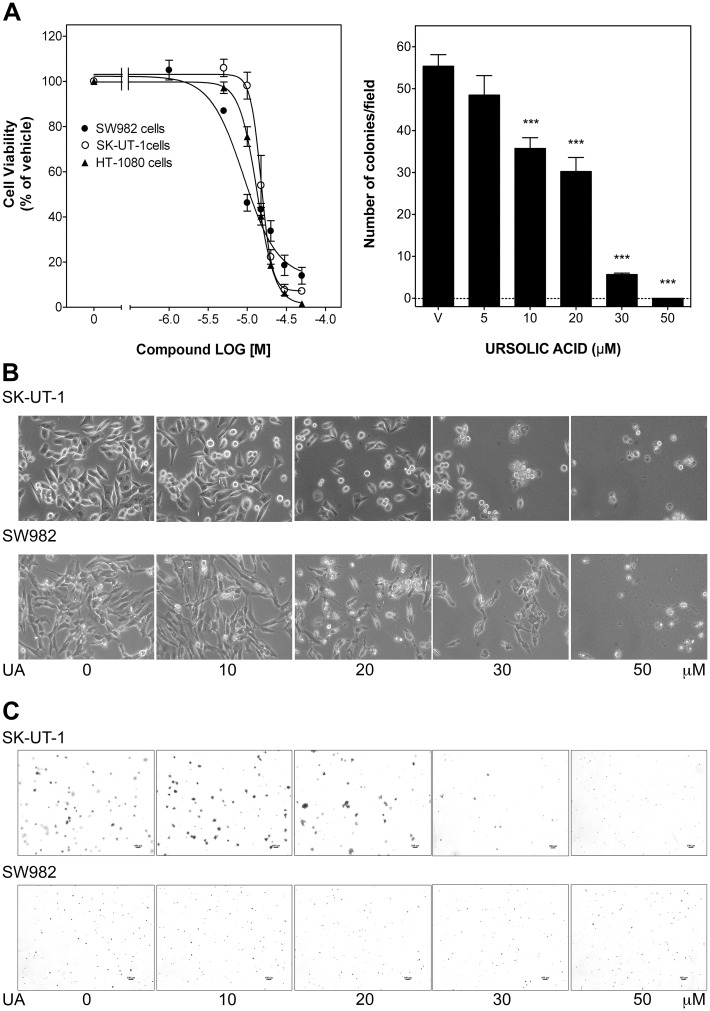
UA dose-dependently inhibited viability and proliferation in human STS cells. A) Fibrosarcoma HT-1080, leiomyosarcoma SK-UT-1 and synovial sarcoma SW982 cells were treated with vehicle (DMSO) or UA (0.1–50 μM) for 24 h. Cell viability was measured by MTT (upper left panel). SK-UT-1 and SW982 cells were cultured in soft agar in the absence (vehicle) or presence of UA (5–50 μM). Columns show only the number of soft agar colonies/field in SK-UT-1 cell cultures, as SW982 cells did not proliferate in semisolid medium (upper right panel). B) Representative images of SK-UT-1 and SW982 cells in the absence (0) or presence of UA (10–50 μM) after 24 h treatment. Magnification 200-fold. C) Representative images of soft agar colonies in SK-UT-1 and SW982 cell cultures in the absence (0) or presence of UA (10–50 μM) after 7 or 21 days, respectively. Magnification 40-fold. Inset bar: 100 μm.

**Fig 2:**
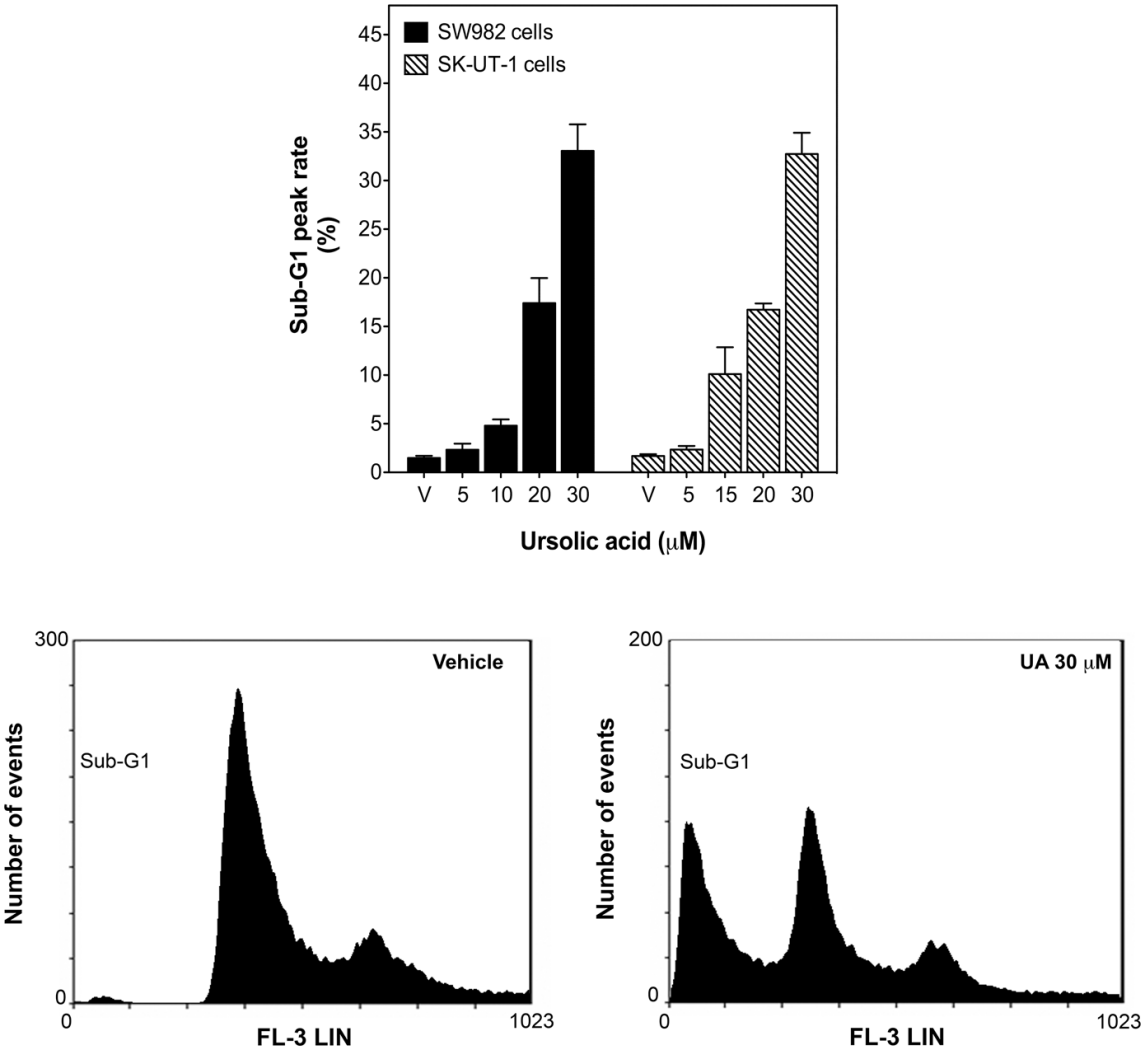
UA dose-dependently induced cell death in human STS cells. Synovial sarcoma SW982 and leiomyosarcoma SK-UT-1 cells were treated with vehicle (DMSO) or UA (5–30 μM) for 24 h. DNA content of cells was measured by flow cytometry. Columns show percentage of apoptotic cells (sub-G1 phase) in the absence (vehicle) or presence of UA. Representative histograms of vehicle-treated and UA-treated (30 μM) SW982 cells are shown below.

Viability and growth inhibition by UA was determined in synovial sarcoma SW982, leiomyosarcoma SK-UT-1 and fibrosarcoma HT-1080 cells. Over a period of 24 h UA inhibited STS cell viability in a concentration-dependent manner and the average IC_50_ values were 9.03 ± 0.04 μM, 15.04 ± 0.02 μM and 13.42 ± 0.01 μM, respectively (Fig 1A), reaching a maximal inhibition of 86% in SW982, 93% in SK-UT-1 and of 100% in HT-1080 cells at 50 μM (Fig 1A and 1B). The strong inhibitory effect of UA at 50 μM on cell viability left only very few cells intact (Fig 1B), which was not sufficient to perform most of the experiments at this concentration. Comparable to the viability results, UA (5–50 μM) also reduced proliferation of SK-UT-1 cells in soft agar with an IC_50_ of 18.33 ± 0.07 μM, reaching a maximal inhibition of 90% and 100% at 30 and 50 μM, respectively (Fig 1A and 1C). Interestingly, SW982 cells were not able to proliferate in semisolid medium at all (Fig 1C), even though the cells were maintained under this condition over a period up to 21 days. Flow cytometry analysis indicated that UA induced a significant cell death up to 17–33% at 20–30 μM for 24 h (Fig 2). As a whole, these data indicate that UA inhibits STS cell viability and proliferation with a predominantly pro-apoptotic effect.

### UA treatment induces intrinsic apoptosis in STS cells

**Fig 3:**
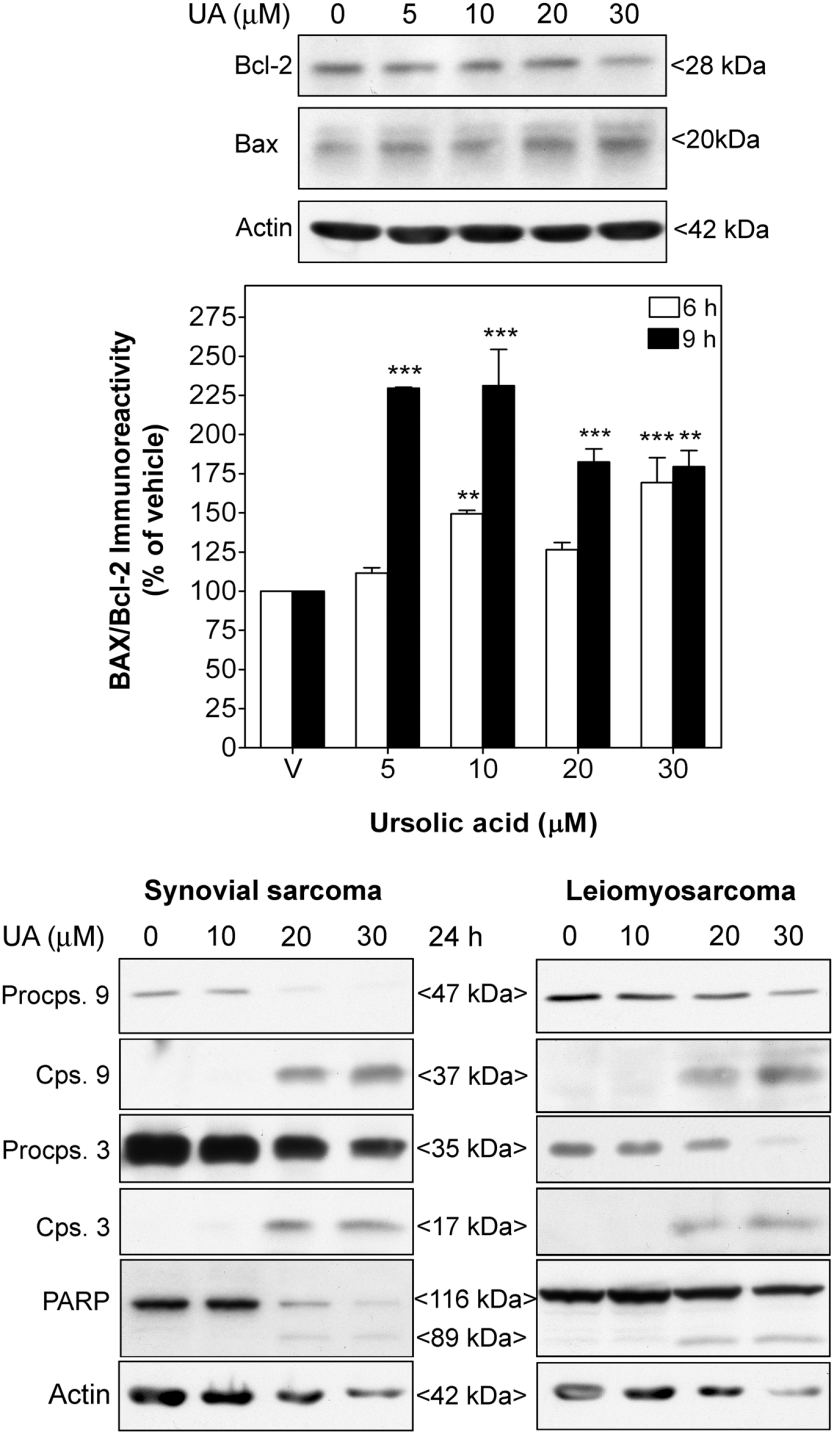
UA induced intrinsic apoptosis in human STS cells. Synovial sarcoma SW982 and leiomyosarcoma SK-UT-1 cells were treated with vehicle (DMSO) or UA (5–30 μM) for 6, 9 and 24 h. *Upper panels* show immunoreactive bands of Bcl-2 and Bax proteins in representative immunoblots of synovial sarcoma SW982 cells treated with UA for 9 h. Columns represent the ratio of the Bax to Bcl-2 immunoreactivity in these cells treated with UA for 6 and 9 h. Each value represents mean ± SEM of 4 independent experiments normalized to vehicle-treated cells (taken as 100%). **P < 0.01 and ***P < 0.001 versus vehicle-treated cells. *Lower panels* show the immunoreactive bands of procaspase 9 and 3, caspase 9 and 3, and PARP fragmentation in representative immunoblots of synovial sarcoma SW982 (left) and leiomyosarcoma SK-UT-1 cells (right) treated with UA for 24 h.

Apoptosis was evaluated by the determination of Bcl-2 and Bax protein expression as well as caspase 3, caspase-9 and PARP processing using western-blot analysis. A decrease in anti-apoptotic Bcl2 expression accompanied by an increase in pro-apoptotic Bax expression was observed in synovial sarcoma cells treated with UA for 6 and 9 h. This led to a significant increase in the Bax/Bcl-2 ratio that reached 2.29 ± 0.01-fold at 5 μM after 9 h (Fig 3). Treatment with UA also provoked dose-dependent processing of procaspase-9 and procaspase-3 at 24 h, inducing the appearance of their active 37-kDa and 17-kDa cleavage fragments, respectively. Dose-dependent cleavage of the caspase-3 substrate PARP was also detected, altogether indicating the activation of intrinsic apoptosis (Fig 3). In contrast to the upper panel of Fig 3, in which treatment was only 9 h to evaluate early events of apoptosis, the lower panel addresses the late events of apoptosis after 24 h. A reduction of actin levels, in particular with high concentrations of UA, could be detected after 24 h, which is most probably caused by the marked effect of UA and the relatively advanced stage of apoptosis.

### UA treatment down-regulates AKT/GSK3β/β-catenin signalling and reduces total c-Myc and p21 levels

**Fig 4:**
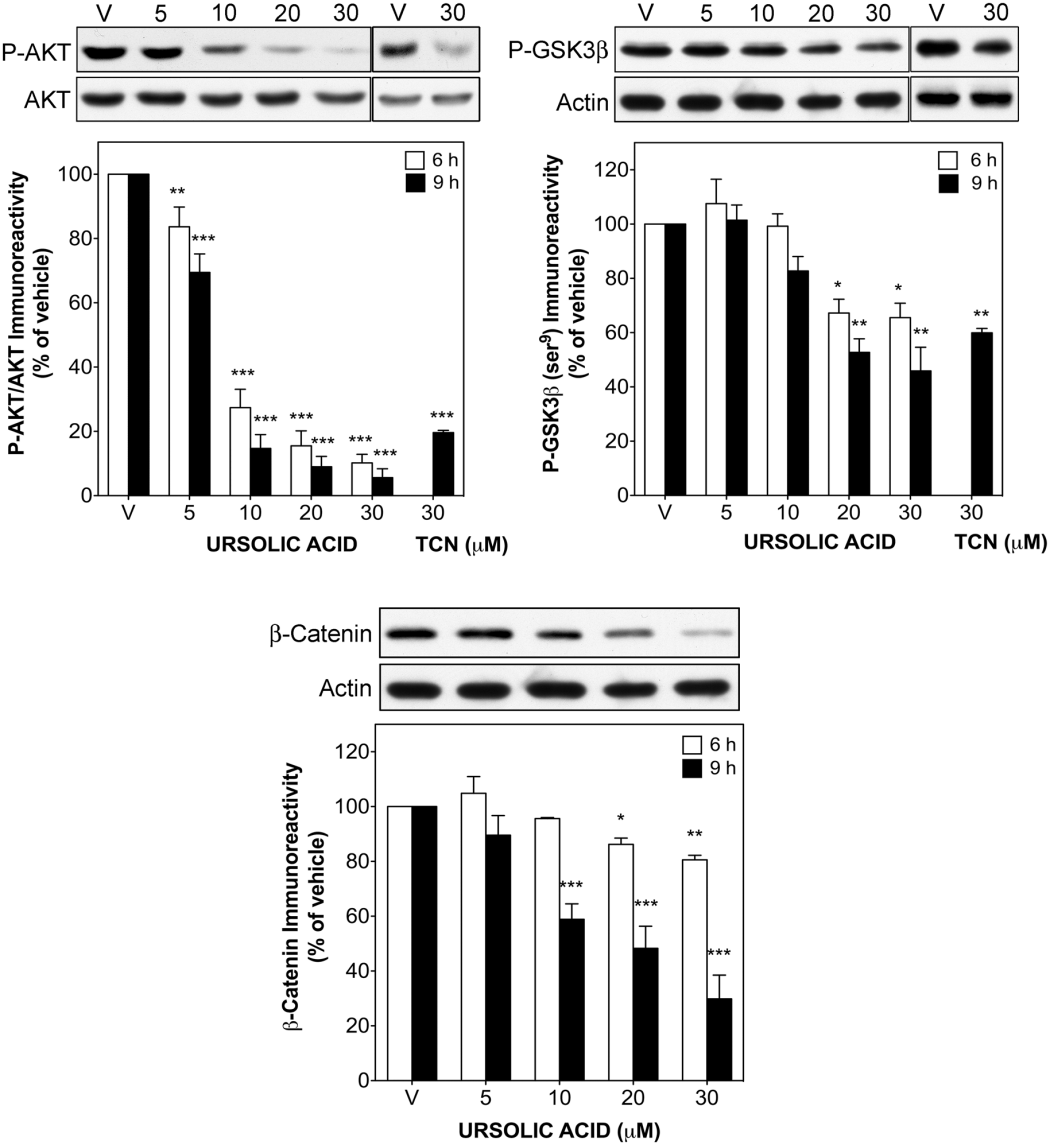
UA down-regulated the AKT/GSK3β/β-catenin pathway in human STS cells. Synovial sarcoma SW982 cells were treated with vehicle (DMSO), TCN (30 μM) or UA (5–30 μM) for 6 and 9 h. *Upper panels* show representative immunoblots of SW982 cells treated with or without UA or TCN for 9 h. Columns represent the phosphorylated ratio of AKT and the ratio of P-GSK3β (ser9) or β-catenin to β-actin immunoreactivity. Each column represents mean ± SEM of 3 independent experiments normalized to vehicle-treated cells (taken as 100%). *P < 0.05, **P < 0.01 and ***P < 0.001 versus vehicle-treated cells.

**Fig 5:**
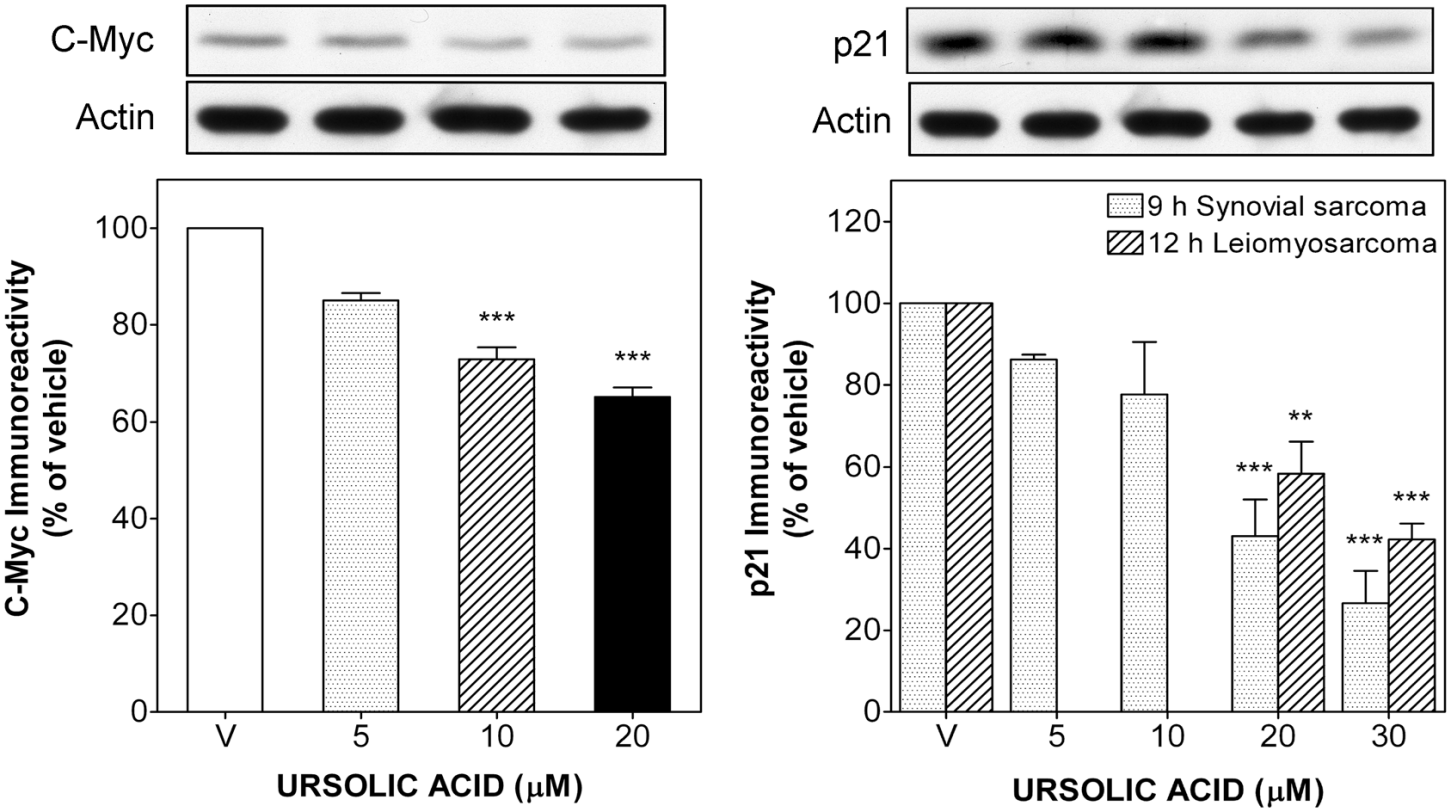
UA down-regulated c-Myc and p21 protein levels in human STS cells. Synovial sarcoma SW982 and leiomyosarcoma SK-UT-1 cells were treated with vehicle (DMSO) or UA (5–30 μM) for 9 or 12 h, respectively. *Upper panels* show immunoreactive bands of c-Myc and p21 proteins in representative immunoblots of SW982 cells treated without or with UA for 9 h. Columns represent the ratio of the c-Myc to β-actin immunoreactivity in SW982 cells (left panel) or p21 to β-actin immunoreactivity in both cell types (right panel). Each value represents mean ± SEM of 3 independent experiments normalized to vehicle-treated cells (taken as 100%). **P < 0.01 and ***P < 0.001 versus vehicle-treated cells.

AKT signalling is an important transduction pathway implicated in controlling proliferation and survival of STS cells, especially in synovial sarcoma [23]. Thus, we evaluated the modulation of the AKT/GSK3β/β-catenin pathway by UA, and its relation to the apoptotic effect of this compound. Short-term treatment with UA (6 and 9 h) drastically decreased phosphorylation of AKT in a dose-dependent manner, reaching a maximal inhibition of 94.4 ± 2.7% in synovial sarcoma (Fig 4) and of 67.6 ± 0.5% in leiomyosarcoma cells (data not shown) at 30 μM in both cases, while total AKT levels remained unchanged. We next analysed the activation state of GSK3β and total levels of β-catenin, both down-stream targets of AKT. UA treatment reduced the inhibitory phosphorylation of GSK3β at ser^9^ as well as total β-catenin levels, reaching an inhibition of 54.3 ± 8.7 and 71.2 ± 8.8% at 30 μM, respectively (Fig 4). Triciribine phosphate (TCN), an AKT inhibitor used as positive control, lowered in a similar way AKT phosphorylation by 80.4 ± 0.7% and, consequently, also reduced the phosphorylation of GSK3β at ser^9^ by 40.1 ± 1.6% at 30 μM (Fig 4). Concomitant with down-regulation of AKT signalling, UA also reduced the expression of the anti-apoptotic proteins c-Myc and p21 (CIP1/WAF1). After 9 h, c-Myc levels decreased 14.9 ± 1.5%, 27.1 ± 2.5% and 34.8 ± 1.9% at 5, 10 and 20 μM, respectively (Fig 5). Likewise, UA (5–30 μM) strongly decreased total p21 levels, reaching a maximal decrease of 73.4 ± 7.9% in synovial sarcoma and 57.8 ± 3.9% in leiomyosarcoma cells after 9 or 12 h of treatment, respectively (Fig 5).

### UA enhances the antitumoral effects of DXR, while in parallel inhibiting DXR-induced AKT activation and p21 expression

In order to investigate whether UA may improve cell susceptibility to DXR, the dose of UA in the combination experiments was adjusted to 10 μM for synovial sarcoma cells and 15 μM for leiomyosarcoma cells, because at these concentrations UA had a similar effect on cell viability and apoptosis in each of the respective cell lines according to their IC_50_ values (Figs 1 and 2). A phase I pharmacokinetic study of UA nanoliposomes showed that plasma concentrations of this compound can reach up to 7.6 μM [27], which similar to the concentrations used here. The concentrations of DXR used in our study (0.1–10 μM) are typical for studies with doxorubicin-resistant or -insensitive cell lines and have been used by us [26] and others in similar *in vitro* studies performed with STS cell lines [28, 29] as well as with other types of cancer cells [30] and can be clinically achieved in human plasma [31].

**Fig 6:**
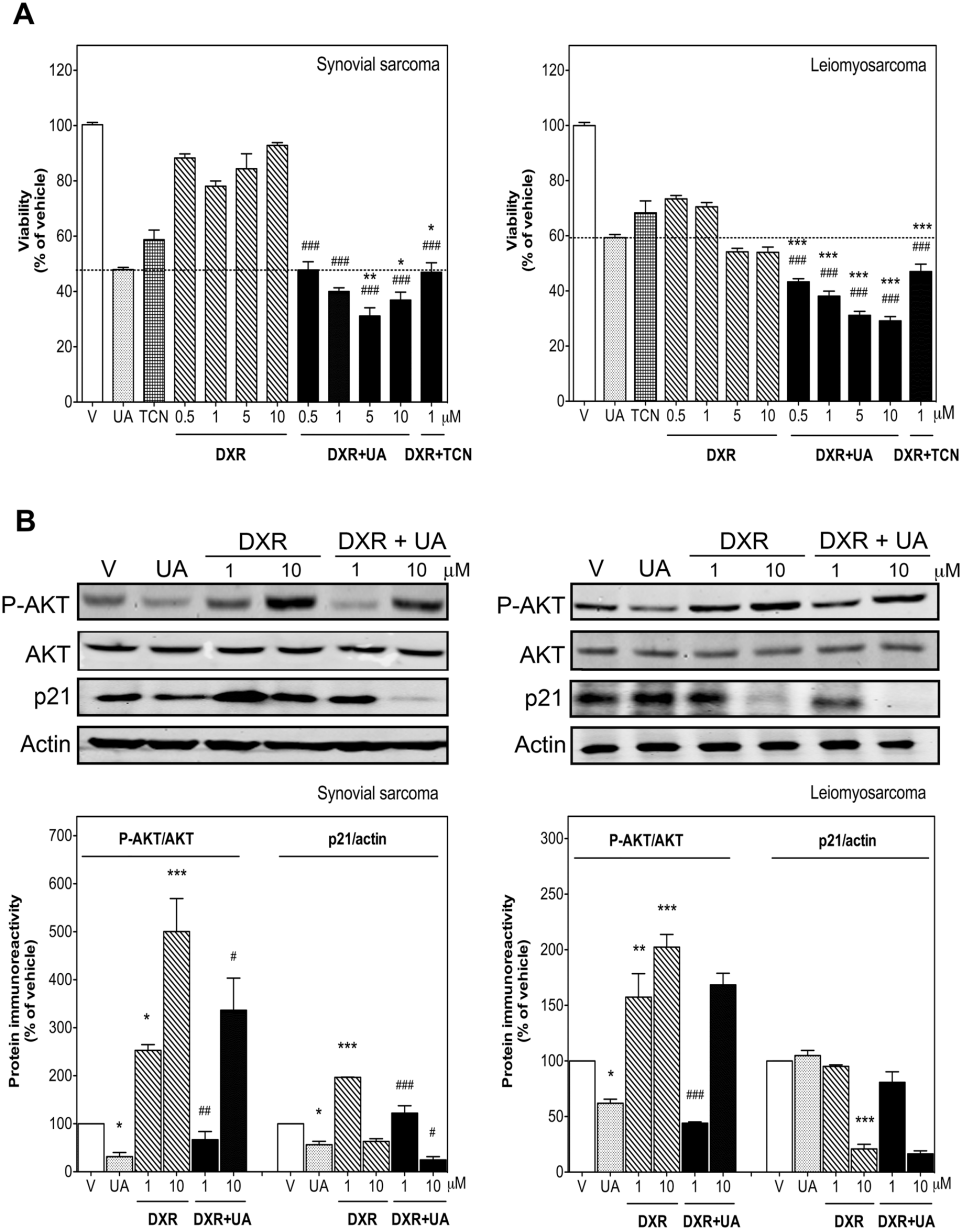
UA enhanced the antitumoral effects of DXR in human STS cells while concomitantly down-regulating DXR-induced AKT phosphorylation and p21 protein levels. A) Synovial sarcoma SW982 (left) and leiomyosarcoma SK-UT-1 cells (right) were treated with vehicle (DMSO) or DXR (0.5, 1, 5 and 10 μM) alone or combined simultaneously with UA (10 μM for SW982 cells or 15 μM for SK-UT-1 cells) or TCN (30 μM) for 24 h. Cell viability was assessed as described in Materials and methods. Each value represent mean ± SEM of 3 independent experiments performed in triplicate and normalized to vehicle-treated cells (taken as 100%). *P < 0.05 and ***P < 0.001 versus UA-treated cells; ^###^*P* < 0.001 versus DXR-treated cells. B) *Upper panels* show representative immunoblots of SW982 cells (left) or SK-UT-1 cells (right) treated with vehicle or DXR (1 and 10 μM) alone or in combination with UA for 9 h. Columns represent the phosphorylated ratio of AKT and the ratio of p21 to β-actin immunoreactivity. Each column represents mean ± SEM of 3 independent experiments normalized to vehicle-treated cells (taken as 100%). *P < 0.05, **P < 0.01 and ***P < 0.001 versus vehicle-treated cells; ^#^*P* < 0.05, ^##^*P* < 0.01 and ^###^*P* < 0.001 versus DXR-treated cells.

As shown in Fig 6A, synovial sarcoma cells were more resistant to the antiproliferative effect of DXR (0.5, 1, 5 and 10 μM) than leiomyosarcoma cells (inhibition of viability between 7.14% and 21.92% and between 15.61% and 45.97%, respectively). However, in both cell lines a higher antiproliferative effect was observed for the combined treatments with DXR and UA than for each compound alone (Fig 6A). Combination of DXR with UA increased the effect of DXR by 2.23- to 8.72-fold in synovial sarcoma cells and by 1.46- to 2.13-fold in leiomyosarcoma cells (the dose response curves for DXR and DXR with UA as well as the corresponding IC_50_ values are included as supplemental data (S1 Fig)). At the molecular level, this potentiation is related to a significant reduction of both DXR-induced AKT phosphorylation and -p21 expression (Fig 6B). Short-term treatments (9 h) with DXR alone at 1 and 10 μM significantly increased AKT phosphorylation by 2.5 ± 0.11-fold and 5.0 ± 0.68-fold in synovial sarcoma cells (Fig 6B) and by 1.57 ± 0.26-fold and 2.0 ± 0.11-fold in leiomyosarcoma cells (Fig 6B). In parallel, expression of p21 protein was also found increased by 1.9 ± 0.01-fold in synovial sarcoma cells but without changes in leiomyosarcoma cells when treated with DXR at 1 μM (Fig 6B). In contrast, at higher concentration (10 μM) DXR significantly reduced total p21 levels by 36.8 ± 5.8% in synovial sarcoma cells as well as by 79.1 ± 4.1% in leiomyosarcoma cells. The combination of low doses of UA with DXR strongly reduced DXR-induced AKT phosphorylation, and further decreased p21 levels, especially in synovial sarcoma cells, thereby enhancing DXR-mediated antiproliferative effects (Fig 6). In agreement with these results, also combination of DXR with TCN (30 μM) increased the effect of DXR by 2.42 ± 0.16-fold in synovial sarcoma cells and by 1.79 ± 0.09-fold in leiomyosarcoma cells (Fig 6A). On the other hand, UA altered neither intracellular DXR concentration (after 24 h intracellular DXR fluorescence in the presence of UA at 10 μM was 96 ± 6% compared to DXR alone) nor uptake of DXR in STS cells (uptake of DXR after 2 h in the presence of UA at 10 μM was 95.9 ± 5.4% compared to DXR alone).

## Discussion

Previous work has suggested that inhibition of AKT could be a desirable therapeutic approach for treatment of STS, in particular, synovial sarcoma. On one hand, activation of the AKT pathway and nuclear β-catenin accumulation has been reported in human synovial sarcoma tissues [23, 24, 32], showing a trend towards poorer survival in those patients with nuclear β-catenin [32, 33]. On the other hand, Bozzi et al. suggested that the overexpression of several tyrosine kinase receptors that signal through AKT contributes to the proliferation of these tumor cells through β-catenin stabilization [23], since mutations in APC (Adenomatous Polyposis Coli) or β-catenin, which would promote its nuclear accumulation, are rare in synovial sarcoma [34, 35]. In addition, a critical role for AKT has also been established in the development of leiomyosarcoma [25].

Although some former studies have linked AKT inhibition to UA-induced suppression of growth and intrinsic apoptosis in certain human carcinoma [10, 36, 37] and leukemia cells [38], our study presents the first evidence that UA is able to suppress proliferation and to induce intrinsic apoptosis through AKT inhibition in human STS cell lines. This is an important finding because STS is a cancer with still few therapeutic options and modern drugs based on specific molecular targeting have not been introduced as standard treatment scheme of STS yet. After short-term treatments (6 or 9 h) with UA a strong AKT inhibition with concomitant GSK3β activation and reduced β-catenin levels could be detected in synovial SW982 sarcoma cells (Fig 4). In this context, TCN, an AKT inhibitor, which also decreased phosphorylation of AKT and GSK3β at ser9 (Fig 4), diminished as well growth in both sarcoma cell lines similar to UA (Fig 6A). The SK-UT-1 cells used in our study differ from SW982 cells as the former have mutations in the *PI3KCA* and *PTEN* genes (phosphatase and tensin homologe, deleted on chromosome 10; http://cancer.sanger.ac.uk/cell_lines/sample/), which should lead to a higher basal AKT activation. Probably as a consequence, UA was still able to induce a significant effect on cell viability, but with lower efficacy compared to the wild type cell line SW982 (Fig 1). Another difference between the cell lines is that UA completely blocked proliferation of SK-UT-1 cells in soft agar, confirming its antiproliferative and apoptotic effect, whereas this effect could not be evaluated in SW982 cells, since they did not grow under soft agar conditions, suggesting an anchorage-dependent proliferation for this sarcoma cell line. Others showed that inhibition of Tcf/β-catenin protein-protein interaction provoked down-regulation of various β-catenin targets, such as c-Myc, and suppressed cell viability in human synovial sarcoma cell lines [39]. These findings are in line with our results, which not only demonstrated a reduced expression of the anti-apoptotic protein c-Myc (a β-catenin target) but also of p21 (Fig 5), which led in a similar manner to reduced cell viability and induction of intrinsic apoptosis (Figs 1 and 2).

PI3K/AKT activation plays an important role in proliferation of cancer cells and also promotes drug resistance by attenuating the antitumoral effect of chemotherapeutics (for a review see [40]). For example, DXR-induced activation of AKT seems to be involved in the resistance of breast cancer cells to this drug [41]. Indeed, our results demonstrate that AKT phosphorylation was also induced in STS cells after only 9 hours of DXR treatment. An increase in AKT phosphorylation may theoretically induce a positive effect on cell viability. However, our results show that the AKT activation by DXR did not increase cell viability compared to control cells. In fact, DXR intercalates between DNA strands, which hinders proliferation. Noteworthy, the antiproliferative effect of DXR alone was relatively weak, especially in synovial sarcoma cells (cell viability decrease of 21.92% at 10 μM) and did not increase in a dose-dependent manner but reached a plateau between 1 and 10 μM after 24 h. These data suggest that AKT phosphorylation may actually represent a compensatory process to counteract the antitumoral effect of DXR. Accordingly, when DXR was combined with UA at concentrations that were able to reduce the DXR-induced AKT activation or with the inhibitor of AKT phosphorylation, TCN, the antiproliferative effect of DXR was significantly increased (Fig 6A).

The cyclin-dependent kinase inhibitor p21 is another essential protein for proliferation, which has been related to cell cycle arrest and apoptosis [42]. Although the role of p21 in apoptosis is complex and depends on the cellular context, it was shown that expression of p21 protected tumor cells from DXR-induced apoptosis [43]. Therefore, it has been suggested that p21 may function as a drug resistance mechanism to chemotherapy-induced apoptosis. Moreover, it has been described that AKT can negatively influence the expression of p21. Our study demonstrates that UA reduces p21 expression in synovial sarcoma cells not only when used as single agent but also in the combined treatment with DXR when compared to DXR alone. Accordingly, UA as single agent was accompanied by apoptosis in both STS cell lines (Fig 3) and also diminished significantly cell viability of DXR treatment when used in combination with this drug (Fig 6).

Oleanolic and maslinic acid, structural analogues of UA, also induce apoptosis in SW982 and SK-UT-1 sarcoma cells and sensitize them to DXR [26]. However, in those cases the sensitization effect was mediated through the selective inhibition of the multidrug resistance (MDR)-related protein 1 (MRP-1) efflux activity, thereby increasing intracellular DXR. In contrast, in our study UA neither altered uptake of DXR nor increased intracellular DXR concentration in STS cells, suggesting that its molecular mechanism of action is different. It has been recently demonstrated in a variety of tumor cell lines that cellular sensitivity to UA was not linked to the expression or activity of P-glycoprotein or MRP-1, implying indirectly that UA, in contrast to oleanolic and maslinic acid, is probably not a substrate of these multidrug resistant proteins and does not interact with them [44]. However, the exact molecular mechanism by which UA inhibits AKT and downstream targets has not been revealed yet. It is well-known that absorption and accumulation of lipids in cell membranes may affect membrane associated signalling events, including AKT activation, and UA could act in a similar way. In this context, perifosine, an alkylphospholipid that introduces into the plasma membrane and blocks AKT phosphorylation and signalling by disrupting AKT translocation to the plasma membrane, has displayed potent antitumor effects not only in human tumor models [45] but also in clinical phase I/II trials, where it was highly efficient in patients with advanced STS, including leiomyosarcomas and synovial sarcomas [46].

In summary, our study reveals that UA not only exhibits a considerable apoptotic effect on STS cells, but also sensitizes them to the current standard chemotherapeutic for this kind of sarcoma, which is DXR. The presented results provide molecular evidence for a possible use of UA in the treatment of certain STS tumors, in particular, synovial sarcoma and leiomyosarcoma. UA is a natural compound present in many dietary components, especially of the Mediterranean diet and, hence, its use as nutraceutical adjuvant together with DXR may ameliorate treatment outcome of patients with STS without increasing the dose of the chemotherapeutic drug or its related toxicity.

## Supporting Information

S1 Fig UA reduced the IC_50_ with which DXR inhibited the viability of human STS cells. Synovial sarcoma SW982 and leiomyosarcoma SK-UT-1 cells were treated with vehicle (DMSO) or doxorubicin (DXR) (range 0.1–10 μM) alone or combined simultaneously with UA (10 μM for SW982 cells or 15 μM for SK-UT-1 cells) for 24 h. Cell viability was assessed as described in Materials and methods. Each value represents mean ± SEM of 3 independent experiments performed in triplicate. The compound concentration resulting in 50% inhibition of cell viability (IC_50_) was determined using GraphPad software. *IC_50_ values of UA were taken from Fig 1. **In SW982 cells a 50% inhibition of cell viability could not be achieved with the maximal concentration of DXR used in our study (10 μM). For this reason, an accurate IC_50_ value could not be calculated. Nevertheless, according to the achieved data points in our experimental setting the IC_50_ value must be higher than 10 μM.
(TIF)
