# Supplementary figures and images for: Down-Regulation of AKT Signalling by Ursolic Acid Induces Intrinsic Apoptosis and Sensitization to Doxorubicin in Soft Tissue Sarcoma
Source: PLoS One. 2016 May 24;11(5):e0155946. doi: 10.1371/journal.pone.0155946 (PMC4878803; doi:10.1371/journal.pone.0155946)

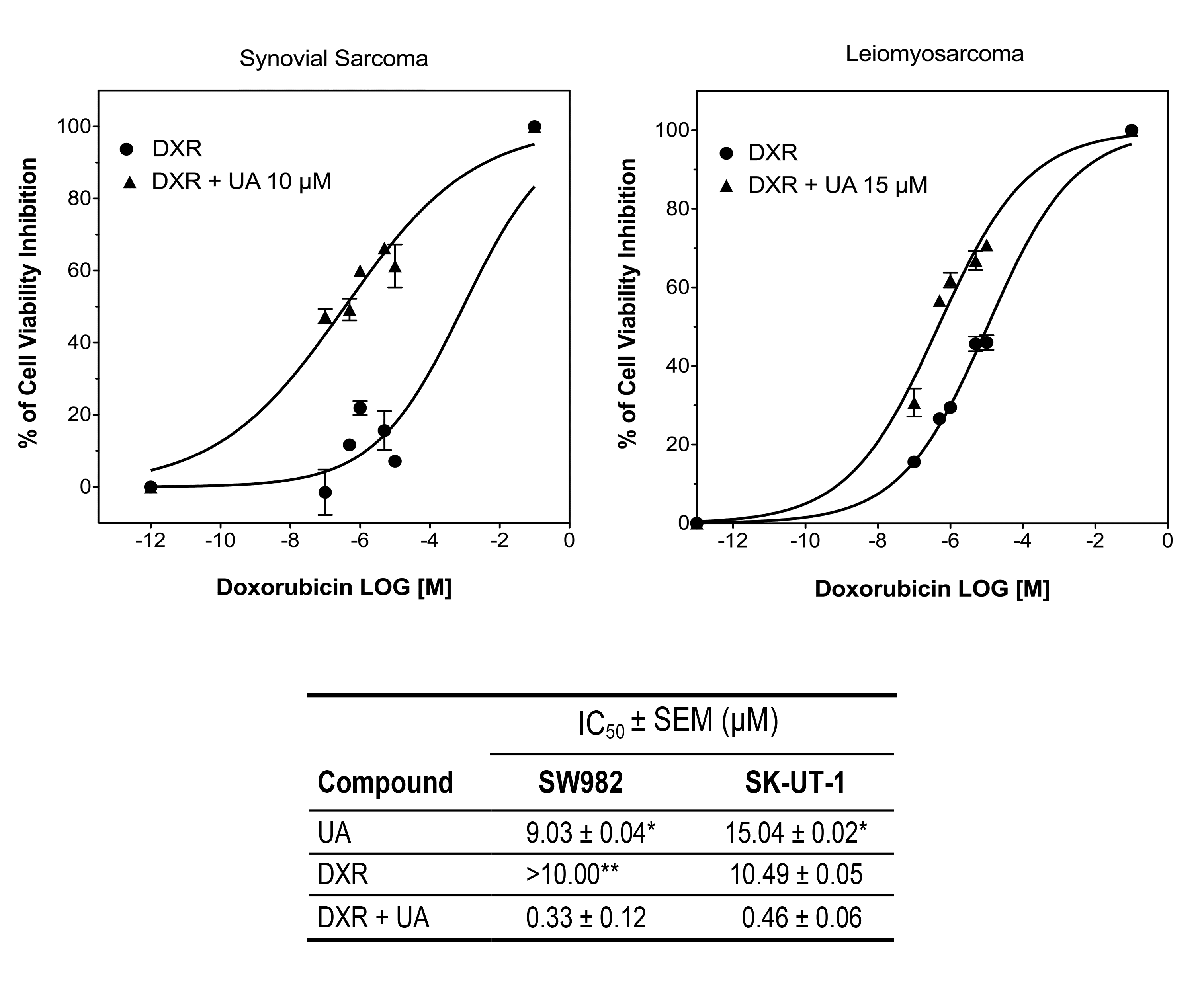

Supplement: S1 Fig — Synovial sarcoma SW982 and leiomyosarcoma SK-UT-1 cells were treated with vehicle (DMSO) or doxorubicin (DXR) (range 0.1–10 μM) alone or combined simultaneously with UA (10 μM for SW982 cells or 15 μM for SK-UT-1 cells) for 24 h. Cell viability was assessed as described in Materials and methods. Each value represents mean ± SEM of 3 independent experiments performed in triplicate. The compound concentration resulting in 50% inhibition of cell viability (IC50) was determined using GraphPad software. *IC50 values of UA were taken from Fig 1. **In SW982 cells a 50% inhibition of cell viability could not be achieved with the maximal concentration of DXR used in our study (10 μM). For this reason, an accurate IC50 value could not be calculated. Nevertheless, according to the achieved data points in our experimental setting the IC50 value must be higher than 10 μM. (TIF) [file pone.0155946.s001.tif]
